# An Exploration of Gene-Gene Interactions and Their Effects on Hypertension

**DOI:** 10.1155/2017/7208318

**Published:** 2017-05-31

**Authors:** Ying Meng, Susan Groth, Jill R. Quinn, John Bisognano, Tong Tong Wu

**Affiliations:** ^1^School of Nursing, University of Rochester, Rochester, NY, USA; ^2^Department of Internal Medicine, Cardiology Division, University of Rochester Medical Center, Rochester, NY, USA; ^3^Department of Biostatistics and Computational Biology, University of Rochester, Rochester, NY, USA

## Abstract

Hypertension tends to perpetuate in families and the heritability of hypertension is estimated to be around 20–60%. So far, the main proportion of this heritability has not been found by single-locus genome-wide association studies. Therefore, the current study explored gene-gene interactions that have the potential to partially fill in the missing heritability. A two-stage discovery-confirmatory analysis was carried out in the Framingham Heart Study cohorts. The first stage was an exhaustive pairwise search performed in 2320 early-onset hypertensive cases with matched normotensive controls from the offspring cohort. Then, identified gene-gene interactions were assessed in an independent set of 694 subjects from the original cohort. Four unique gene-gene interactions were found to be related to hypertension. Three detected genes were recognized by previous studies, and the other 5 loci/genes (*MAN1A1*, *LMO3*, *NPAP1/SNRPN*, *DNAL4*, and *RNA5SP455/KRT8P5*) were novel findings, which had no strong main effect on hypertension and could not be easily identified by single-locus genome-wide studies. Also, by including the identified gene-gene interactions, more variance was explained in hypertension. Overall, our study provides evidence that the genome-wide gene-gene interaction analysis has the possibility to identify new susceptibility genes, which can provide more insights into the genetic background of blood pressure regulation.

## 1. Introduction

Hypertension (HTN) affects 1 out of 3 adults in the United States and is a major risk factor for stroke and cardiovascular diseases (CVDs) [1]. Antihypertensive therapy has considerably reduced the morbidity and mortality associated with CVDs [2]. However, nearly half of hypertensive patients do not have their blood pressure (BP) under control [1]. Thus, alternative strategies and more individualized treatments for BP reduction are particularly desirable.

Evidence has suggested that the heritability of HTN is around 20–60% [3–5]. Genome-wide association studies (GWASs), especially a recent meta-analysis with large consortia, have been productive in recognition of hypertension susceptibility genes, and more than 200 loci have been identified [6–15]. But the genetic contribution accounted for by the identified variants is limited [5–7]. A large amount of genetic variance is still left unexplained, which is referred to as “missing heritability.”

Several new approaches have been explored to search for the missing genetic contribution. Gene-gene (GxG) interactions, also called epistasis, is one approach that has the potential to enlighten the missing heritability [16]. Biologically, the regulation of BP consists of complex physiological pathways, which include the cardiovascular, neural, renal, endocrine system and local tissue [17]. Genes that are involved in the function of these systems interact with each other to maintain the homeostasis of BP. Therefore, hypertension is probably the outcome of multiple genes acting conjointly. Statistically, single-locus GWASs and meta-analyses have been focused on identification of genes with main effects. An epistasis analysis, on the other hand, is capable of discovering genes with nonapparent or weak effects, when the GxG interactions are significant [18, 19]. Consequently, studies of GxG interactions have the potential to recognize novel genes that may be missed in meta-analyses if the main genetic effects are minimal and to contribute to the explanation of the heritability of hypertension.

To date, a few genome-wide analyses have been conducted to identify GxG interactions for hypertension [19–23]. These studies have identified new genes in addition to the findings of single-locus GWASs, which further supports the value of the epistasis analysis. Interestingly, most genes identified by these epistasis analyses had no main effects [19–22]. However, only one of these studies has replicated their findings in an additional population [22], and the sample size in that study was relatively small (350 subjects) at the discovery stage, which potentially limited their findings. For the studies without appropriate replications, the identified GxG interactions could not be verified, and other possible explanations, such as population structure, could not be ruled out. In addition, none of the above studies examined the variance in HTN that could be accounted for by the identified GxG interactions, which would substantiate whether GxG interactions contribute to the explanation of the missing heritability.

For the current study, we explored the contribution of GxG interactions to HTN using a two-stage approach, the discovery stage and the confirmatory stage. We incorporated the boolean operation-based screening and testing (BOOST) method developed by Wan et al. [19] to detect GxG interactions at the discovery stage. The BOOST method is an effective epistasis approach especially for detecting GxG interactions when there are no main effects [24] and has been frequently adopted to conduct an epistasis analysis [21, 23, 25–27]. Following the discovery stage, the interactions were verified in a separate sample at the confirmatory stage. The purpose of the current study was to identify hypertension susceptibility GxG interactions on the basis of a two-stage approach including a genome-wide discovery analysis and a confirmatory association analysis. Furthermore, the contribution of GxG interactions was also assessed with regard to the heritability of hypertension.

## 2. Materials and Methods

### 2.1. Framingham Heart Study Samples

We used the data from the Framingham Heart Study [FHS; https://www.ncbi.nlm.nih.gov/projects/gap/cgi-bin/study.cgi?study_id=phs000007.v28.p10]. FHS is a longitudinal cohort study that was initiated in 1948 to investigate the common risk factors for CVDs. The study has collected a vast array of phenotypes including BP periodically over three generations: the original cohort, the offspring cohort, and the third-generation cohort [4, 28]. In the current study, a two-stage analysis was performed. The genome-wide discovery scan was conducted in 2320 cases with matched controls from the offspring cohort. The following confirmatory association analysis was applied to 694 cases with matched controls from the original cohort. The third generation was not included due to the limited period over which the phenotypes were measured. The current study was approved by the Institutional Review Board.

### 2.2. Blood Pressure Phenotypes

The original cohort examinations 1–17 and the offspring cohort examinations 1–8 were included in this study. At each clinical examination, the systolic and diastolic BP (SBP and DBP) were measured three times by two physicians and a nurse/technician. The BP measurements from the physicians were averaged to generate the SBP and DBP readings for that examination. The BP measurements from the nurse/technician were used if the measurements from one physician were missed. If the BP measurements were missed from both physicians, the observations were removed from that examination. For participants taking antihypertensive medications, 10 mmHg and 5 mmHg were added to the SBP and DBP values, respectively [11].

The inclusion criteria for the case groups were defined as follows: (1) SBP ≥ 140 mmHg by two physicians' measures or (2) DBP ≥ 90 mmHg by two physicians' measures or (3) taking antihypertensive medications. The participants who fulfilled the inclusion criteria at any examination were selected as the hypertensive cases. Participants were considered candidates for the controls if their BPs were below 130/80 mmHg without taking BP-lowering medications and they were not allocated to cases (hypertensive) at any examination. The exclusion criteria for both case and control groups were as follows: (1) age > 65 years or (2) body mass index (BMI) ≥ 30 kg/m^2^ or (3) potential secondary causes of hypertension, including thyroid disorders and renal diseases, or (4) diabetes mellitus. Cases did not undergo screening to exclude secondary HTN. Additionally, individuals whose reported sex did not match the genomic data were removed from the sample. Finally, a one-to-one matching based on propensity scores was applied to balance age, sex, BMI, and examination [29] between the cases and controls in both cohorts. Hypertension was used as the primary outcome. BP traits (SBP and DBP) were used as the secondary outcomes.

### 2.3. Genotyping Methods

Affymetrix 550K genotyping data (GeneChip Human Mapping 500K Array Set and HuGeneFocused 50K Array) from the Framingham Heart Study were utilized in the current study. Single-nucleotide polymorphisms (SNPs) were filtered according to these exclusion criteria: genotyping call rate for each SNP < 95%, minor allele frequency (MAF) < 1%, and Hardy-Weinberg equilibrium *p* value <1 × 10^−6^. Individuals with an overall genotyping call rate less than 95% were also excluded. In total, there were 438,565 SNPs available for the subsequent analysis. In addition, population stratification in each cohort was assessed, and the first 10 principal components (PCs) were generated and used as covariates.

### 2.4. Statistical Methods

At the discovery stage, the genome-wide GxG interaction scan was conducted using the BOOST method [19]. In the BOOST test, all pairwise interactions were first examined using Kirkwood superposition approximation which compares the joint distribution under the full logistic regression model with the joint distribution under the main-effect model. Interactions that passed a specified threshold (*p* = 4.89 × 10^−6^) were further assessed using the likelihood ratio statistic and the *χ*^2^ test to compute the interaction effects and the significance levels of the interactions. The top 100 unique GxG interactions with the lowest *p* values were selected based on the results from the BOOST test. These top 100 interactions were further examined by linear regression (for SBP and DBP) and logistic regression (for HTN) which were adjusted for covariates, including age, sex, BMI, and 10 PCs. The significance level for the regression analyses was set at 5 × 10^−4^ after Bonferroni correction.

At the confirmatory stage, interactions significant in either regression analysis were verified in the FHS original cohort. Linear and logistic regressions adjusted for covariates were used again to evaluate the candidate interactions. Two criteria were used to justify the HTN susceptibility GxG interactions, including *p* < 0.05 and the interaction effects on BP/HTN consistent between the original and offspring cohorts.

Single-locus associations of the SNPs in the top 100 unique interactions were assessed using the linear and logistic regressions adjusted for covariates in the offspring cohort to examine their main effects on the BP traits and HTN. Haplotype blocks were also estimated for these SNPs to determine the uniqueness of the GxG interactions. Interactions formed by SNPs from the same haplotype block were considered the same interaction.

Phenotype data were processed using STATA 13.0 (StataCorp, College Station, TX). The BOOST test, linear regression of the interactions, linear and logistic regressions of the single locus, and haplotype block estimation were conducted using PLINK1.9 (https://www.cog-genomics.org/plink2; [30, 31]). Logistic regression of the interactions was completed using CASSI to control covariates [Howey; http://www.staff.ncl.ac.uk/richard.howey/cassi/].

## 3. Results

### 3.1. The Discovery Stage

Characteristics of the participants from the offspring cohort are shown in S1 Table available online at https://doi.org/10.1155/2017/7208318. There were 1246 males and 1074 females. The mean age in the cases was 49 years old, which was around 2.6 years older than the controls (*p* < 0.001). The difference in BMI was relatively small (mean difference = 0.5, *p* < 0.001). Sex was exactly matched between cases and controls. The range of SBP was from 99 to 209 mmHg in the cases and from 86 to 130 mmHg in the controls. The range of DBP was from 63 to 125 mmHg in the cases and from 50 to 80 mmHg in the controls.

First, the exhaustive pairwise genome-wide GxG interaction analysis was performed using the BOOST test. The quantile-quantile plot for the interaction analysis on HTN is presented in S1 Figure. 145 SNP pairs were selected, which represented the top 100 unique SNP pairs with the lowest significance levels because some SNPs were in linkage disequilibrium. Then, these SNP pairs were further evaluated using the linear and logistic regressions, since age and BMI were not perfectly matched between cases and controls. Also, the population structure was adjusted in the regression analyses. Totally, 71 unique SNP pairs (103 SNP pairs) were significant after Bonferroni correction (*p* < 5 × 10^−4^) for either HTN or BP traits (S1 Dataset).

### 3.2. The Confirmatory Stage

The cases and controls from the FHS original cohort were selected according to the same criteria as for the offspring cohort. Characteristics of the subjects from the original cohort are listed in S2 Table. The proportion of females in this sample was higher compared to that of the offspring cohort. The difference in mean age between the cases and controls was smaller (*p* = 0.5995) than seen in the offspring cohort. The difference in BMI was similar to that of the offspring sample (*p* = 0.0096). The range of SBP in the sample from the original cohort was from 115 to 203 mmHg in the cases and from 84 to 130 mmHg in the controls. The range of DBP was from 54 to 115 mmHg in the cases and from 57 to 80 mmHg in the controls.

The 71 unique GxG interactions identified at the discovery stage were assessed with the sample from the original cohort. Six unique SNP pairs remained significant (*p* < 0.05, S2 Dataset). Among them, two SNP pairs were dropped because their effects on DBP were not consistent between the original cohort and the offspring cohort. The remaining four unique GxG interactions (5 SNP pairs) are presented in Table 1. The majority of SNPs in the interactions are intron variants, except for two SNPs, rs3913226 and rs17688362, which are located outside of currently known gene regions. The nearest genes to these two SNPs were searched for and obtained from the National Center for Biotechnology Information (NCBI) human genome database (annotation release 107; http://www.ncbi.nlm.nih.gov/projects/genome/guide/human/index.shtml).

Single-locus associations of the SNPs identified at the discovery stage were also conducted on the BP traits and HTN in the offspring cohort. Three genetic models, additive, dominant, and recessive, were adopted in the association analyses. The main effects of the SNPs which were significant at the confirmatory stage are presented in S3 Dataset. None of the SNPs were significantly related to the BP traits or HTN. The *p* values ranged from 0.0598 to 0.9805.

The contribution of the four unique GxG interactions was also assessed using linear and logistic regressions. In the offspring cohort, the main effects of the SNPs in these GxG interactions together explained 0.04% to 0.56% of the variance in SBP, DBP, and HTN. The GxG interactions alone (main effect was not included), however, explained 3.98% to 4.86% of the variance in the BP traits and HTN. In the original cohort, the accumulated main effects of the SNPs were ranged from 0.04% to 1.66%. By including the interactions, the variance explained in BP and HTN was also improved, though the magnitude was decreased to 0.33% to 1.32% (main effect was not included).

### 3.3. Interaction Patterns

The interaction patterns for the four distinct gene pairs in the offspring sample are presented in Table 2. The interaction found between rs4235144 and rs9489622 is located within the gene gamma-aminobutyric acid type A receptor beta1 subunit (GABRB1) and the gene mannosidase alpha class 1A member 1 (*MAN1A1*). The SNPs have a MAF of 0.015 and 0.49, respectively. The genotypes CT/GA and CT/AA demonstrated a protective effect (odds ratio (OR) = 0.36 and 0.07, resp.) from developing hypertension compared to the homozygous TT/AA. The genotype CT/GG appeared to be overrepresented in the hypertensive group. The interaction patterns were further assessed in the original sample (S3 Table). None of the genotypes for this interactive SNP pair were significant due to the limited sample size. But the directions of these genotypes were similar between the two cohorts (S3a Table). The other SNP pairs (rs4235144-rs9481890) within the same GxG interaction presented a similar pattern.

The interaction between rs2058798 and rs3913226 involves a rhombotin family of cysteine-rich LIM domain oncogene (*LMO3*) and a gene encoding nuclear pore-associated protein 1 (*NPAP1*) or a gene encoding small nuclear ribonucleoprotein polypeptide N (*SNRPN*). The MAFs of these SNPs are 0.012 and 0.18, respectively. The genotype AG/CC had a higher odds of hypertension (OR = 28.30; 95% CI: 3.836–208.829), while the genotype AG/TC had a lower odds (OR = 0.28; 95% CI: 0.103–0.743). The confidence intervals are broader for these genotypes because the MAF of rs2058798 is relatively rare, and consequently, the individuals who carried these two genotypes are limited. The directions of the majority genotypes, nonetheless, were replicated in the original sample (S3b Table).

The interaction between rs1909884 and rs17284390 is located within the gene cholinergic receptor nicotinic alpha 7 subunit (*CHRNA7*) and the gene cadherin 13 (*CDH13*). The MAFs of these SNPs are 0.36 and 0.11, respectively. For the genotype pairs AA/AA, GG/GG, and GG/GA), the odds of developing hypertension were lower compared to those for the most common double homozygous GG/AA. The ORs ranged from 0.03 to 0.63. The genotype AA/GA, however, had a higher risk for hypertension (OR = 1.72; 95% CI: 1.029–2.877). The directions of the majority genotypes were consistent with those in the original cohort (S3c Table).

The interaction between rs17688362 and rs12484954 involves the RNA 5S ribosomal pseudogene 455 (*RNA5SP455*)/keratin 8 pseudogene 5 (*KRT8P5*) and the gene dynein axonemal light chain 4 (*DNAL4*). The two SNPs are relatively common (MAFs of 0.21 and 0.39, resp.). Overall, the odds of hypertension were increased by carrying minor alleles, except for the genotype TT/CG which had a lower odds ratio (Table 2(d), OR = 0.27; 95% CI: 0.128–0.564). When compared to the interaction pattern in the original sample, the directions for the majority genotype pairs were comparable (S3d Table).

## 4. Discussion

Compared to single-locus GWASs, a GxG interaction analysis is capable of identifying susceptibility genes with weak or no main effects [18, 19]. Therefore, additional susceptibility genes can be recognized using this methodology as was done in the current study. Among the genes identified in the four unique GxG interactions, none of them had a significant main effect on BP or HTN in the FHS population. Two of the genes, *CDH13* and *GABRB1*, have been associated with hypertension in a previous GWAS [6, 7, 15]. The gene *CHRNA7*, though it has not been identified by other GWASs, has been shown to play a role in BP regulation in rat models [32–34]. The remaining 5 loci/genes are new findings.

The gene *MAN1A1* encodes a type of *α*l,2-mannosidases located in the Golgi [35]. In humans, *MAN1A1* was first isolated from the kidney and was also observed in other tissues and organs, such as the liver, spleen, and leukocytes [36, 37]. The function of *MAN1A1* has not been investigated extensively. One study has found the product enzyme was involved in immune function and played a role in T cell activation [38]. Interestingly, another study has shown that the activity of *α*-mannosidase in the urine was increased in patients with severe gestational hypertension during pregnancy [39].

Two other identified genes, *LMO3* and *SNRPN*, are predominantly expressed in the brain [40, 41]. Although they have not been directly linked to hypertension, both have interactions with the tumor suppressor protein p53 (*TP53*). *LMO3* has been found to interact with *TP53* directly and repress *TP53*-dependent mRNA expression [42]. The interaction between *SNRPN* and *TP53* was recognized by the two-hybrid interaction mating [43]. *TP53* is a well-known tumor suppressor gene that regulates the transcription of diverse target genes and is involved in a variety of cellular functions. Evidence suggests that *TP53* contributes to BP regulation and hypertension-induced complications, such as left ventricular hypertrophy [44–46].

In addition, *LMO3* also promotes adipogenesis, and studies have shown that adipose tissue participates in the renin-angiotensin system, which is a well-recognized BP regulation system [47, 48]. Interestingly, rs3913226 (between *NPAP1* and *SNRPN*) is located within chromosome 15q11–13, a region associated with Prader-Willi syndrome. One characteristic of Prader-Willi syndrome is hyperphagia with obesity [49]. Therefore, it is also possible that *LMO3* and *SNRPN* (or *NPAP1*) participate in BP regulation through another mechanism, such as adipose tissue.

The gene *DNAL4* encodes a dynein light chain which is a component of the dynein motor complex [50]. In humans, *DNAL4* is highly expressed in the testis, thyroid, and brain. This gene has been suggested to play a role in the neurotrophin signaling pathway which is essential for the proper function and survival of neurons (REACTOME: R-HSA-177504). Also, a study has found that the dynein motor complex participates in the cytoplasmic transportation of *TP53* [51]. The final two identified genes, *RNA5SP455* and *KRT8P5*, are pseudogenes, whose function has not been discovered.

The four GxG interactions discovered in this study are all novel discoveries. None of the previously identified GxG interactions were replicated in this study. A possible explanation is that epistasis analyses are typically low yield (0-1 SNP pair identified per study) due to the strict Bonferroni correction for multiple testing [19–21]. The challenge of multiple testing is more prominent in epistasis analyses compared to single-locus GWASs. For example, there are approximately 125 billion SNP pairs to be tested in a GWAS with 500,000 SNPs. A *p* value less than 10^−13^ is rare for a GWAS at this scale after Bonferroni correction. Another less conservative approach, false discovery rate, was also adopted to select genome-wide significant GxG interactions [22]. The number of significant outcomes was improved slightly but still limited (one SNP pair identified at the confirmatory stage). Other methods, such as gene-based interaction analyses, have the potential to enhance the power to identify more GxG interactions, if the computational efficacy is adequate to conduct genome-wide analyses [52]. Meanwhile, an epistasis meta-analysis also has the capability of improving the findings and replications.

As indicated by previous studies, an interaction found by an epistasis analysis does not mean that the two genes are connected with each other directly [20–22]. The two genes may be involved in the same pathway or have a similar impact on a disease trait. None of the GxG interactions identified in the current study are interactive directly. However, mutual proteins were identified by consulting the Biological General Repository for Interaction Datasets (https://thebiogrid.org/) and the Human Protein Reference Database (http://www.hprd.org/index_html) for protein-protein interactions. For example, *TP53* has a connection to the gene pair, *LMO3* and *SNRPN*. For two other interactions, *GABRB1* and *MAN1A1* and *CHRNA7* and *CDH13*, a network of multiple genes instead of a single gene may establish the relationship between the two genes. For instance, the possible linkages can be plotted as *GABRB1-PIK3CA-PIK3R1-TYK2-PLAUR-MAN1A1* and *CHRNA7*-*APP*-*APOA1*-*MAPK6*-*CDH13* according to the protein-protein interaction network.

Our results indicate that the explanation of the heritability of BP was improved by including GxG interactions. In single-locus GWASs, the amount of the variation in BP traits explained by the identified SNPs together has typically been less than 3% in individual studies [6, 7, 9, 14]. The variance explained in our study by the four identified GxG interactions (main effects were not included) was comparable to this amount. It is possible that as more GxG interactions are discovered, a greater amount of heritability can be explained. On the other hand, a proportion of the heritability of BP may be accounted for by rare alleles [9], epigenetic factors, such as DNA methylation at promoter sites to regulate the expression of hypertension-related genes [53], and gene-environmental interactions, such as gene-sodium interactions [54].

In summary, our study identified new GxG interactions using the genome-wide epistasis analysis, and the findings were confirmed with an additional sample. The limitation of our study is the relatively small sample size. For future studies, replication with larger samples and other populations is desirable to investigate the roles of these newly identified genes and interactions. Overall, our study further demonstrates the ability of a genome-wide GxG interaction analysis in detection of novel genes which are potentially missed in single-locus GWASs due to weak or no main effects on phenotypes. Moreover, our study supports the hypothesis that GxG interactions can contribute to the explanation of the missing heritability of hypertension.

## Supplementary Material

Supporting Information. S1 Fig. Quantile-quantile plots of the BOOST test statistic observed for hypertension. S1 Table. Summary of Characteristics of Case and Control Groups from the Offspring Sample. S2 Table. Summary of Characteristics of Case and Control Groups from the Original Sample. S3 Table. Genotype Counts for SNP Pairs and Odds Ratios Relative to the Double Major Allele Homozygote Genotype in the Original Sample. S1 Dataset. Significant GxG interactions during the discovery stage. S2 Dataset. Significant GxG interactions during the discovery stage and the confirmatory stage. S3 Dataset. Single-locus GWAS under different genetic models for SNPs identified in the GxG interaction analyses.

## Figures and Tables

**Table 1:** Significant gene-gene interactions in both the discovery stage and the confirmatory stage.

CHR1	SNP1	MA1	CHR2	SNP2	MA2	P_BOOST	P_SBP	B_SBP	P_DBP	B_DBP	P_HTN	LOR_OR_HTN	GENE1	GENE2
4	rs4235144	C	6	rs9489622	G	6.55E-10	0.0063	24.35			0.0155	16.43	GABRB1	MAN1A1
4	rs4235144	T	6	rs9481890	T	6.41E-10	0.0067	24.20			0.0153	16.12	GABRB1	MAN1A1
12	rs2058798	A	15	rs3913226^∗^	T	9.38E-10					0.0391	−16.72	LMO3	NPAP1/SNRPN
15	rs1909884	A	16	rs17284390	G	3.38E-10			0.0479	2.54			CHRNA7	CDH13
18	rs17688362^∗^	T	22	rs12484954	C	5.21E-10			0.0436	−2.03	0.0057	−0.56	RNA5SP455/KRT8P5	DNAL4

*Note*. CHR represents chromosome. SNP is single-nucleotide polymorphism. MA means minor allele. P_BOOST is the *p* value of the BOOST test in the offspring sample. P_SBP is the *p* value of the linear regression on systolic blood pressure in the original sample. P_DBP is the *p* value of the linear regression on diastolic blood pressure in the original sample. P_DBP is the *p* value of the logistic regression on hypertension in the original sample. ∗ indicates the SNP is located outside of the gene region.

**(a):** 

		*rs9489622*		
	rs4235144	*GG*	*GA*	*AA*
Controls	CC	0	0	0
	CT	0	18	14
	TT	278	550	292
Hypertension	CC	0	0	0
	CT	17	7	1
	TT	262	556	315
OR^a^ relative to TT/AA (95% CI)	CC	—	—	—
	CT	—	0.36 (0.148–0.876)^∗^	0.07 (0.009–0.507)^∗^
	TT	0.87 (0.693–1.102)	0.94 (0.769–1.142)	1

**(b):** 

		*rs3913226*		
	rs2058798	*TT*	*TC*	*CC*
Controls	AA	0	0	0
	AG	2	19	1
	GG	25	310	781
Hypertension	AA	0	0	0
	AG	0	5	27
	GG	30	332	745
OR^a^ relative to GG/CC (95% CI)	AA	—	—	—
	AG	0.21 (0.010–4.375)	0.28 (0.103–0.743)^∗^	28.30 (3.836–208.829)^∗^
	GG	1.26 (0.733–2.159)	1.12 (0.934–1.350)	1

**(c):** 

		*rs17284390*		
	rs1909884	*GG*	*GA*	*AA*
Controls	AA	2	24	132
	AG	8	117	398
	GG	15	108	351
Hypertension	AA	7	46	82
	AG	15	110	428
	GG	0	76	391
OR^a^ relative to GG/AA (95% CI)	AA	3.14 (0.648–15.225)	1.72 (1.029–2.877)^∗^	0.56 (0.409–0.761)^∗^
	AG	1.68 (0.705–4.018)	0.84 (0.627–1.137)	0.97 (0.792–1.177)
	GG	0.03 (0.002–0.486)^∗^	0.63 (0.456–0.876)^∗^	1

**(d):** 

		*rs12484954*		
	rs17688362	*CC*	*CG*	*GG*
Controls	TT	3	40	8
	TG	60	198	121
	GG	88	325	307
Hypertension	TT	12	9	26
	TG	57	160	149
	GG	109	363	257
OR^a^ relative to GG/GG (95% CI)	TT	4.78 (1.334–17.117)^∗^	0.27 (0.128–0.564)^∗^	3.88 (1.728–8.723)^∗^
	TG	1.13 (0.762–1.691)	0.97 (0.740–1.260)	1.47 (1.099–1.969)^∗^
	GG	1.48 (1.068–2.050)^∗^	1.33 (1.067–1.668)^∗^	1

*Note*. ^∗^*p* < 0.05. ^a^OR is odds ratio.
