# Supplementary material for: An Exploration of Gene-Gene Interactions and Their Effects on Hypertension
Source: Int J Genomics. 2017 May 31;2017:7208318. doi: 10.1155/2017/7208318 (PMC5470022; doi:10.1155/2017/7208318)
Supplement: Supplementary file 1 — Supporting Information. S1 Fig. Quantile-quantile plots of the BOOST test statistic observed for hypertension. S1 Table. Summary of Characteristics of Case and Control Groups from the Offspring Sample. S2 Table. Summary of Characteristics of Case and Control Groups from the Original Sample. S3 Table. Genotype Counts for SNP Pairs and Odds Ratios Relative to the Double Major Allele Homozygote Genotype in the Original Sample. S1 Dataset. Significant GxG interactions during the discovery stage. S2 Dataset. Significant GxG interactions during the discovery stage and the confirmatory stage. S3 Dataset. Single-locus GWAS under different genetic models for SNPs identified in the GxG interaction analyses. [file 7208318.f1.pdf]

Supplementary Figure 1. **Quantile-quantile plots of the BOOST test statistic observed for hypertension**. Only interactions with *p* values ≤ 1×10^{-4} were obtained due to the extensive numbers of the epistasis tests conducted. In order to compare the observed values to the expected ones, the *p* values of all interactions were divided by 1×10^{-4}.

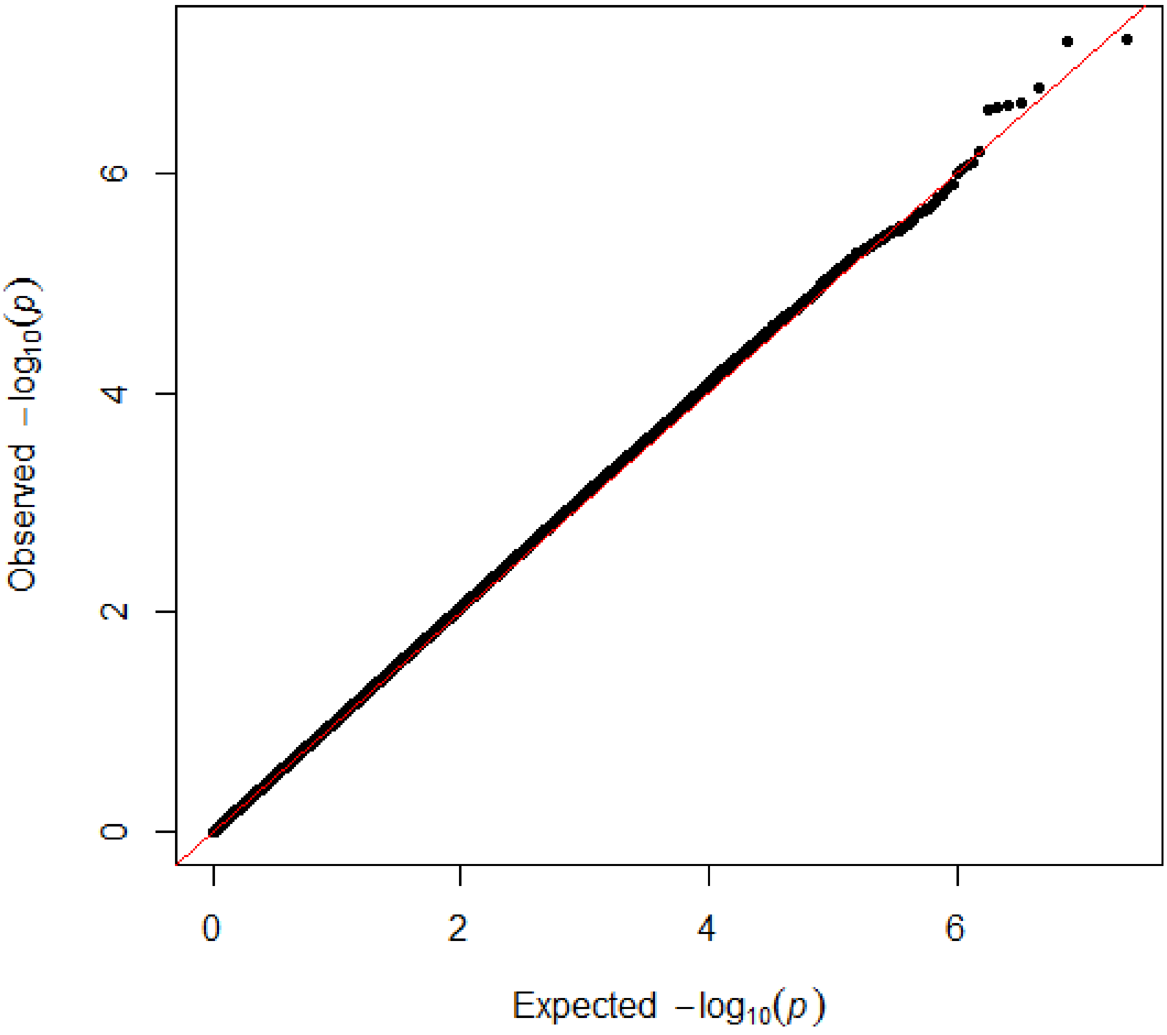

S1 Table. Summary of Characteristics of Case and Control Groups from the Offspring Sample

	Case	Control
**Age**	49.1(±9.17)	46.5(±9.13)
**Sex(male)**	53.7%	53.7%
**BMI**	25.8(±2.67)	25.3 (±2.59)
**SBP**	143.0(±14.60)	112.7(±7.97)
**DBP**	89.4(±8.98)	71.7(±5.48)

The numbers presented in the table are the means and standard deviations except for the item of sex. The numbers for sex are the percentage of males in either group. The standard deviations are in the () beside the means.

S2 Table. Summary of Characteristics of Case and Control Groups from the Original Sample

	Case	Control
**Age**	49.0(±7.63)	48.7(±7.40)
**Sex(male)**	37.2%	37.2%
**BMI**	25.0(±2.72)	24.5 (±2.69)
**SBP**	144.0(±12.08)	114.1(±8.42)
**DBP**	89.3(±8.01)	72.5(±5.53)

The numbers presented in the table are the means and standard deviations except for the item of sex. The numbers for sex are the percentage of males in either group. The standard deviations are in the () beside the means.

S3 Table. Genotype Counts for SNP Pairs and Odds Ratios Relative to the Double Major Allele Homozygote Genotype in the Original Sample

S3a.

			rs9489622	
	rs4235144	*GG*	*GA*	*AA*
Controls	CC	0	0	0
	CT	0	5	2
	TT	74	185	80
Hypertension	CC	0	0	0
	CT	2	5	0
	TT	82	174	84
OR[a] relative to TT/AA (95% CI)	CC	-	-	-
	CT	-	0.95(0.266-3.415)	-
	TT	1.06(0.681-1.637)	0.90(0.619-1.296)	1

S3b.

			rs3913226	
	rs2058798	*TT*	*TC*	*CC*
Controls	AA	0	0	0
	AG	0	1	6
	GG	12	80	245
Hypertension	AA	0	0	0
	AG	0	0	10
	GG	12	95	217
OR[a] relative to GG/CC (95% CI)	AA	-	-	-
	AG	-	0.38(0.015-9.285)	1.88(0.673-5.263)
	GG	1.13(0.497-2.565)	1.34(0.946-1.901)	1

S3c.

			rs17284390	
	rs1909884	*GG*	*GA*	*AA*
Controls	AA	0	13	40
	AG	2	32	105
	GG	4	32	118
Hypertension	AA	0	13	33
	AG	0	29	120
	GG	0	23	127
OR[a] relative to GG/AA (95% CI)	AA	-	0.93(0.414-2.086)	0.77(0.454-1.295)
	AG	0.19(0.009-3.912)	0.84(0.480-1.476)	1.06(0.739-1.526)
	GG	0.10(0.006-1.939)	0.67(0.370-1.207)	1

S3d.

	rs17688362	*rs12484954* *CC*	*CG*	*GG*
Controls	TT	4	5	2
	TG	26	50	37
	GG	38	93	78
Hypertension	TT	1	8	5
	TG	11	44	53
	GG	39	101	73
OR[a] relative to GG/GG (95% CI)	TT	0.27(0.029-2.446)	1.71(0.535-5.465)	2.67(0.503-14.199)
	TG	0.45(0.209-0.980)*	0.94(0.561-1.575)	1.53(0.903-2.594)
	GG	1.10(0.633-1.899)	1.16(0.758-1.776)	1

* $P < 0.05$. [a]OR is odds ratio.
